# *In vivo* tracking of human placenta derived mesenchymal stem cells in nude mice *via*^14^C-TdR labeling

**DOI:** 10.1186/s12896-015-0174-4

**Published:** 2015-06-13

**Authors:** Cheng-Guang Wu, Ji-Chun Zhang, Cheng-Quan Xie, Ornella Parolini, Antonietta Silini, Yi-Zhou Huang, Bing Lian, Min Zhang, Yong-Can Huang, Li Deng

**Affiliations:** Laboratory of Stem Cell and Tissue Engineering, Regenerative Medicine Research Center, West China Hospital, Sichuan University, Chengdu, People’s Republic of China; Centro di Ricerca E.Menni, Fondazione Poliambulanza, Brescia, Italy; West China School of Pharmacy, Sichuan University, Chengdu, People’s Republic of China; Center Laboratory For Isotopy, West China Hospital, Sichuan University, Chengdu, People’s Republic of China; Department of Orthopaedics and Traumatology, The University of Hong Kong, Hong Kong, SAR People’s Republic of China; Laboratory of Stem Cell and Tissue Engineering, State Key Laboratory of Biotherapy, West China Hospital, Sichuan University, Chengdu, People’s Republic of China

## Abstract

**Background:**

In order to shed light on the regenerative mechanism of mesenchymal stem cells (MSCs) *in vivo*, the bio-distribution profile of implanted cells using a stable and long-term tracking method is needed. We herein investigated the bio-distribution of human placental deciduas basalis derived MSCs (termed as PDB-MSCs) in nude mice after intravenous injection by carbon radioisotope labeling thymidine (^14^C-TdR), which is able to incorporate into new DNA strands during cell replication.

**Results:**

The proliferation rate and radioactive emission of human PDB-MSCs after labeled with different concentrations of ^14^C-TdR were measured. PDB-MSCs labeled with 1 μCi possessed high radioactivity, and the biological characteristics (*i.e.* morphology, colony forming ability, differentiation capabilities, karyotype and cell cycle) showed no significant changes after labeling. Thus, 1 μCi was the optimal concentration in this experimental design. In nude mice, 1 × 10^6^^14^C-TdR-labeled PDB-MSCs were injected intravenously and the organs were collected at days 1, 2, 3, 5, 30 and 180 after injection, respectively. Radiolabeled PDB-MSCs were found mainly in the lung, liver, spleen, stomach and left femur of the recipient nude mice at the whole observation period.

**Conclusions:**

This work provided solid evidence that ^14^C-TdR labeling did not alter the biological characteristics of human placental MSCs, and that this labeling method has potential to decrease the signal from non-infused or dead cells for cell tracking. Therefore, this labeling technique can be utilized to quantify the infused cells after long-term follow-up in pre-clinical studies.

## Background

Mesenchymal stem cells (MSCs) have been regarded as a promising candidate for cell therapy with increasing evidence of improved therapeutic effectiveness in various diseases [1, 2]. However, the fate and the role of MSCs in disease progression and regression remain largely unclear. In *vivo* tracking of implanted MSCs will provide a powerful tool to investigate the MSCs-mediated regenerative mechanism; especially, the survival, migration and differentiation of MSCs are crucial for successful regeneration. Thus, reliable and quantitative tracking methods to monitor the *in vivo* bio-distribution of MSCs are highly desirable in animal studies.

Many methods for stem cells tracking are available currently, such as histological detection of xenogeneic or chemical-labeled cells, magnetic resonance imaging scans (MRI), quantum dots (QDs) using fluorescent semiconductor nanocrystals, ultrasound and radiolabeling by nuclear medicine technology [3–8]. Most of them are capable of real-time observation and noninvasive, but the observation time is restricted owing to quick signal strength decline, limiting the accuracy and reliability of experimental results.

Radiolabeled thymidine, such as tritiated thymidine (^3^H-TdR) and ^14^C-TdR, can be taken up by the replicating cells and is commonly used for cell cycle analysis to measure the cell proportion at S phase. Due to the long half-life (12.35 years) and high radioactivity, ^3^H-TdR has been used to detect the dynamic distribution of MSCs [9]. Similarly, ^14^C has a prominent long half-life (about 5730 years) which guarantees the long-term and stably radioactive signal for *in vivo* detection. Additionally, thymidine labeling in the 2-carbon position can directly degrade into labeled CO_2_ rather than labeled β-aminoisobutyric acid or other subsequent metabolites which might be absorbed by the host cells, thus disturbing the accuracy of detection [10–13]. Hence, these advantages of ^14^C-TdR ensure its potential application in cell tracking to aid in long-term observation after stem cells grafting.

Human placenta-derived MSCs, similar to those from other sources, possess *in vitro* differentiation and immunomodulatory capacities [14]. Moreover, when transplanted in preclinical models, placenta-derived MSCs show mostly anti-inflammatory and anti-fibrotic effects [15]. In previous study, we have successfully isolated MSCs form the deciduas basalis of placenta (termed as PDB-MSCs) [16]. In order to test the feasibility of ^14^C labeled thymidine as the potential tracker for MSCs tracking and to investigate the bio-distribution of PDB-MSCs after implantation, PDB-MSCs was labeled with ^14^C-TdR and then injected into the nude mice *via* the caudal vein for bio-distribution study.

## Results

### Determination of the optimal ^14^C-TdR labeling concentration for PDB-MSCs

**Fig. 1:**
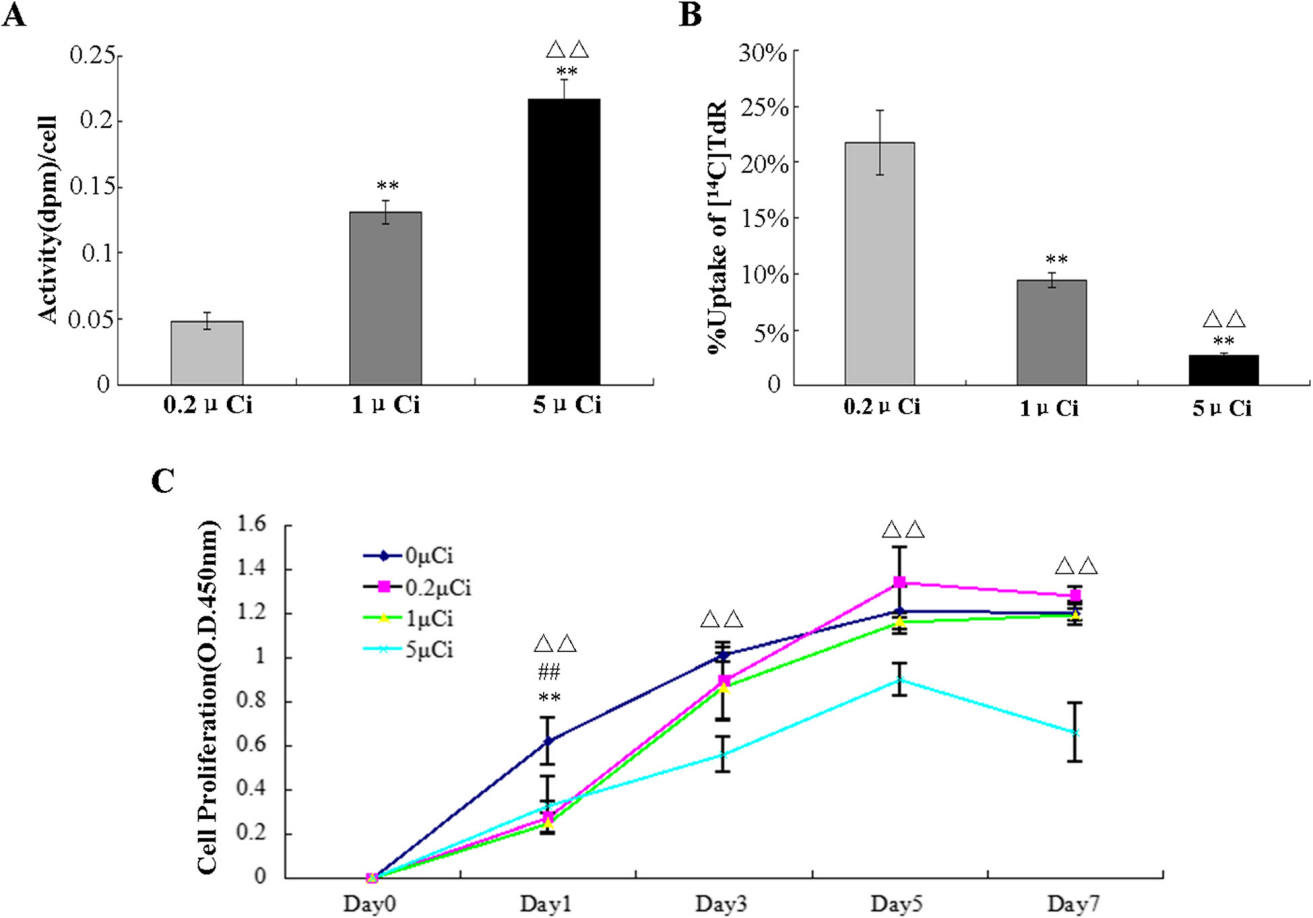
Optimization of ^14^C-TdR labeling concentration for PDB-MSCs. **a** In *vitro* cellular uptake of ^14^C-TdR in three different concentrations (0.2, 1 and 5μCi). Disintegration per minute (dpm) per cell was used to express the radioactivity. The radioactivity per cell increased in a concentration-dependent manner (n = 5). The radioactivity per cell increased in a concentration-dependent manner (n = 5). **b** ^14^C-TdR percentage uptake decreased with the concentration (n = 5). **P < 0.01 compared with the 0.2μCi group; ^ΔΔ^ *P* < 0.01 compared with the 1 μCi group. **c** The proliferation curve of the 4th passage of PDB-MSCs: 5μCi ^14^C-TdR labeled PDB-MSCs group showed a lower growth rate than any other groups. One μCi and 0.2μCi groups had the similar proliferation ability compared to the unlabeled group (n = 6). Data is shown as mean ± SD, N = 5 for ^14^C-TdR labeling study, N = 6 for cell proliferation analysis. ^**^ *P* < 0.01 1 μCi group compared with the control group; ^##^ *P* < 0.01 0.2μCi group compared with the control group; ^ΔΔ^ *P* < 0.01 5μCi group compared with the control group

To determine the optimal concentration, the labeling efficiency of different concentrations of ^14^C-TdR was analyzed after incubation with PDB-MSCs for 72 h. As shown in Fig. 1a, the uptake of ^14^C-TdR, represented as disintegrations per minute (dpm) per cell, increased in a dose-dependent manner; unlabeled, 0.2, 1 and 5 μCi-labeled groups resulted in 0, 0.0481 ± 0.006, 0.131 ± 0.009 and 0.217 ± 0.015 dpm/cell, respectively (Fig. 1a). However, increasing concentrations of ^14^C-TdR administration were associated with the decreasing of total uptake efficiency (determined as the percentage of absorbed ^14^C-TdR versus total treatment dose) of PDB-MSCs because of limited absorption ability; 0.2 μCi, 1 μCi and 5 μCi treated groups resulted in 21.7 ± 2.88 %, 9.45 ± 0.66 % and 2.75 ± 0.19 % uptake, respectively (Fig. 1b). Moreover, the effect of different concentrations of ^14^C-TdR on PDB-MSCs proliferation was investigated and CCK-8 was used to assess cell growth rate. As shown in Fig. 1c, there was no statistical difference in the growth rate between the control group and those labeled with 0.2 and 1 μCi from day 3 to day 7 (*P* > 0.05 vs. control). However, a significant inhibition of proliferation was recorded in the group labeled with 5 μCi with the lowest growth rate during the 7-day period (*P* < 0.01 vs. control). These findings suggested that radiotoxicity occurred when the dose was higher than 1 μCi.

Hence, considering the long-term safety and labeling sensitivity, even though both 0.2 μCi and 1 μCi did not inhibit the cellular proliferation, 1 μCi was the optimal concentration for PDB-MSCs labeling owing to its higher radioactivity (0.131 ± 0.009 dpm/cell). This concentration would increase the signal intensity during detection and thus was used in the subsequent experiments.

### Biological characteristics of ^14^C-TdR-labeled PDB-MSCs

In order to understand the effects of ^14^C-TdR labeling on the biological characteristics of PDB-MSCs, the morphology, differentiation, colony-forming abilities, karyotype, and cell cycle were analyzed.

**Fig. 2:**
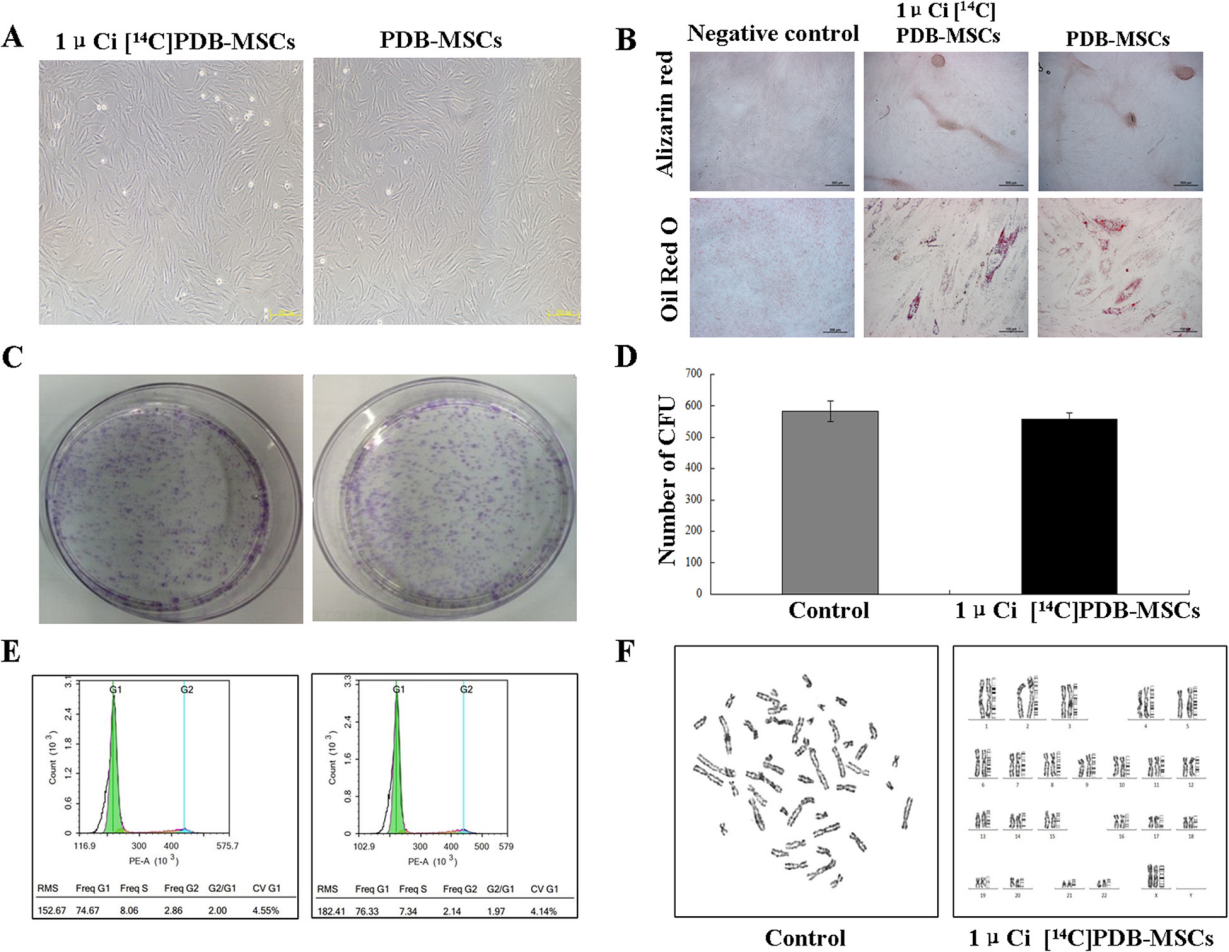
Analysis of biological characteristics of PDB-MSCs after labeling. **a** The 4th passage of PDB-MSCs showed similar fibroblast-like morphology after cultivation in standard culture medium with and without 1 μCi ^14^C-TdR for 72h. Scale bar = 200μm. Differentiation capability of 1 μCi ^14^C-TdR labeled and unlabeled PDB-MSCs (**b**). Alizarin Red staining of differentiated (day 21) and control cells for osteogenic differentiation study, scale bar = 500μm. Oil red O staining of differentiated (day 8) and control cells for adipogenic differentiation study, scale bar =100μm. The CFU potential of 1 μCi ^14^C-TdR labeled and unlabeled PDB-MSCs at 8 days (**c** and **d**). Crystal violet (0.1 %) staining of 1 μCi ^14^C-TdR labeled (557 ± 20.1) and unlabeled (583 ± 32.5) PDB-MSCs shows no significant difference in colony-forming ability (n = 4) (*P* > 0.05). The 4th passage PDB-MSCs stained with PI were prepared for cell cycle analysis by NovoCyte flow cytometer and data were analyzed by NovoExpress analysis software. 1 μCi ^14^C-TdR labeled PDB-MSCs and unlabeled PDB-MSCs showed the similar trend and proportion of G0/G1, S and G2-M phase (**e**). Chromosome spread and karyotypic analysis of 1 μCi ^14^C-TdR labeled 4th passage PDB-MSCs (**f**). 22 pairs of somatic chromosomes were shown, as well as two X chromosomes, for a total of 46 chromosomes. Data were expressed as mean ± SD, N = 4 for CFU experiments

As shown in Fig. 2a, PDB-MSCs labeled with 1 μCi displayed a similar fibroblastic and spindle-shaped morphology, similar to that of unlabeled PDB-MSCs. After culture with specific induction medium, labeled PDB-MSCs were positive for Alizarin Red and Oil Red O, respectively, while those cultured in regular medium were negative (Fig. 2b). Additionally, there was not significant difference in the numbers of colony forming units (CFU) between the labeled and unlabeled groups: the number of unlabeled group was 583 ± 32.5 and that of the labeled group was 557 ± 20.1 (Fig. 2c&d). After cell cycle analysis (Fig. 2e), it was found that both of labeled and unlabeled PDB-MSCs displayed the highest percentage in G0/G1 phase without significant alteration (labeled group 74.67 %, unlabeled group 76.33 %). Furthermore, 1 μCi ^14^C-TdR labeling did not change the karyotype of PDB-MSCs (Fig. 2f). Thus, the above data indicated that there was no obvious change in PDB-MSCs after 1 μCi ^14^C-TdR labeling.

### Bio-distribution of PDB-MSCs in nude mice

**Fig. 3:**
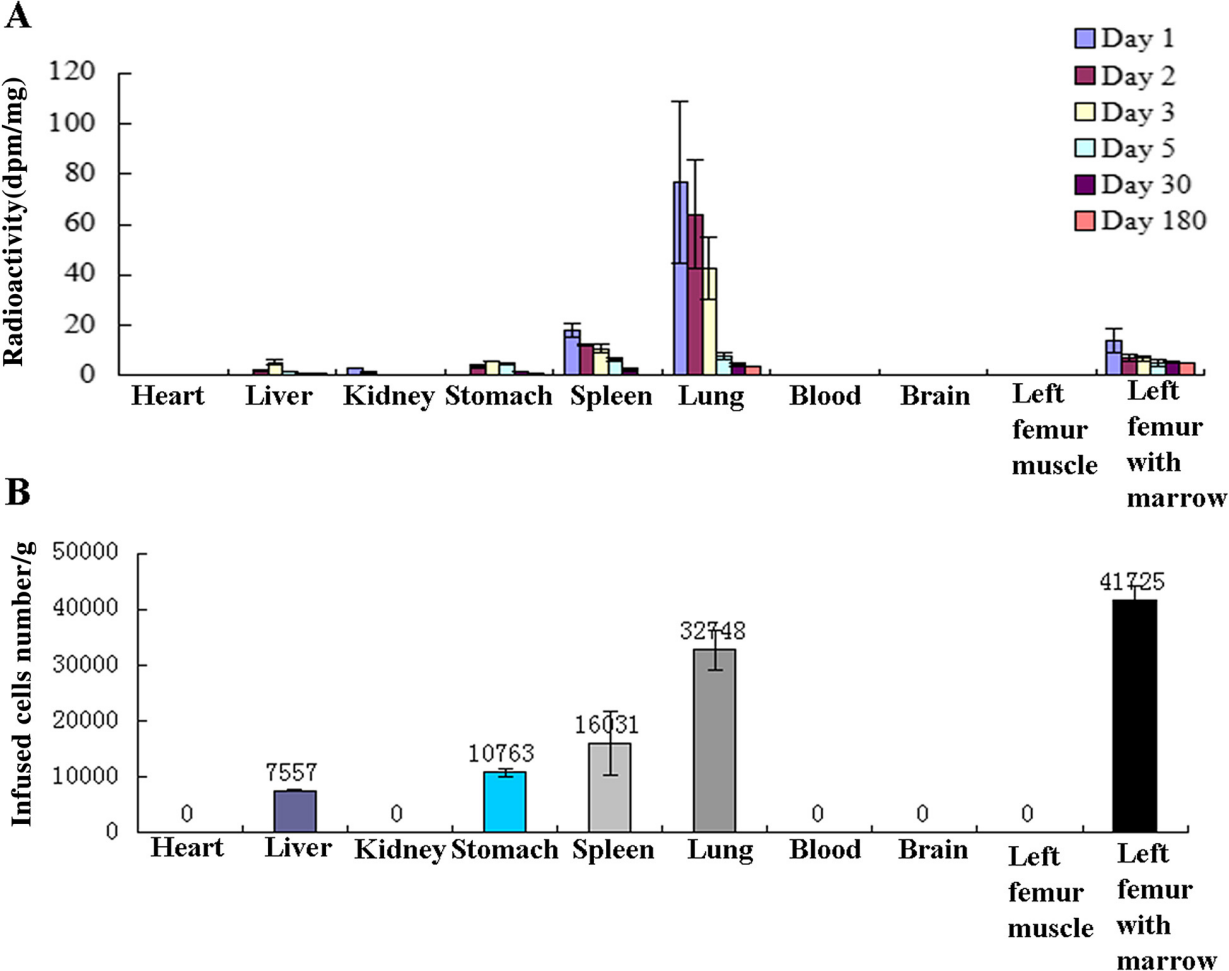
Bio-distribution and quantitative results of infused ^14^C-TdR labeled PDB-MSCs. **a** The bio-distribution of 1 μCi ^14^C-TdR labeled 4th passage PDB-MSCs after IV injection in nude mice. ^14^C radioactivity of each organ was detected by liquid scintillator counter at 6 different time points after injection (at day 1, day 2, day 3, day 5, day 30 and day 180). **b** Quantification of infused ^14^C-labeled PDB-MSCs in different organs of the nude mice on day 30. The quantity of infused cells is expressed as number per gram which is calculated through the average activity of 1 μCi labeling PDB-MSCs (0.131dpm/cell) and the radioactivity (dpm/mg) of the organs at day 30 (**a**). Values were presented as mean ± SD of three independent experiments

At days 1, 2, 3, 5, 30 and 180 post injection, the radioactivity of each organ was measured following decay and background corrections, and data were represented as mean dpm/mg ± standard deviation (SD). At day 1 after injection, radioactivity was highest in the lung (76.7 ± 31.9 dpm/mg) and it was detectable in the kidney, spleen, left femur and bone marrow (Fig. 3a); but it was not detected in the organs from the control group (radioactivities of the organs from control group had no significant difference with the background radioactivity). And the radioactivity in liver and stomach were noted at day 2 after injection.

The radioactivity of the kidney, spleen and lung were diminished with time and the signal in the kidney was disappeared on day 3. Dramatically, cell detection in the lung decreased from day 3 to day 5 (42.3 ± 12.3 dpm/mg to 7.64 ± 1.59 dpm/mg) (Fig. 3a). Beyond a decline on day 2, a stable radioactivity was observed in the left femur and bone marrow which was even peaked on day 30 (5.47 ± 0.314 dpm/mg) (Fig. 3a). The radioactivity value of liver increased from 2.05 ± 0.257 dpm/mg (day 2) to 5.31 ± 1.337 dpm/mg on day 3; and then it decreased gradually to 0.99 ± 0.027 dpm/mg on day 30. Similarly, radioactivity value of stomach experienced a rise from 3.55 ± 0.797 dpm/mg (day 2) to 5.74 ± 0.058 dpm/mg on day 3 and dropped to 1.41 ± 0.102 dpm/mg on day 30 (Fig. 3a). No radioactivity was found in the heart, blood, brain and left femur muscle (Fig. 3a).

The number of infused cells in each organ on day 30 was calculated according to average radioactivity of infused cells. As shown in Fig. 3b, the left femur harvested with bone marrow had the highest number of infused PDB-MSCs per gram (41,725 ± 2394/g) and the number of cells in lung, spleen, stomach, and liver detected on day 30 were 32,748 ± 3586, 16,031 ± 5640, 10,763 ± 777, 7557 ± 207 per gram of tissue, respectively. As two mice died because of the feeding machine accident, only one mouse was used for organs collection on day 180. The radioactivity in the organs were mainly detected in liver (1.08 dpm/mg), lung (3.82 dpm/mg) and left femur harvested with bone marrow (5.31 dpm/mg), indicating that the infused PDB-MSCs were able to exist in bone tissue up to 6 months (Fig. 3a).

### Immunohistochemistry results

**Fig. 4:**
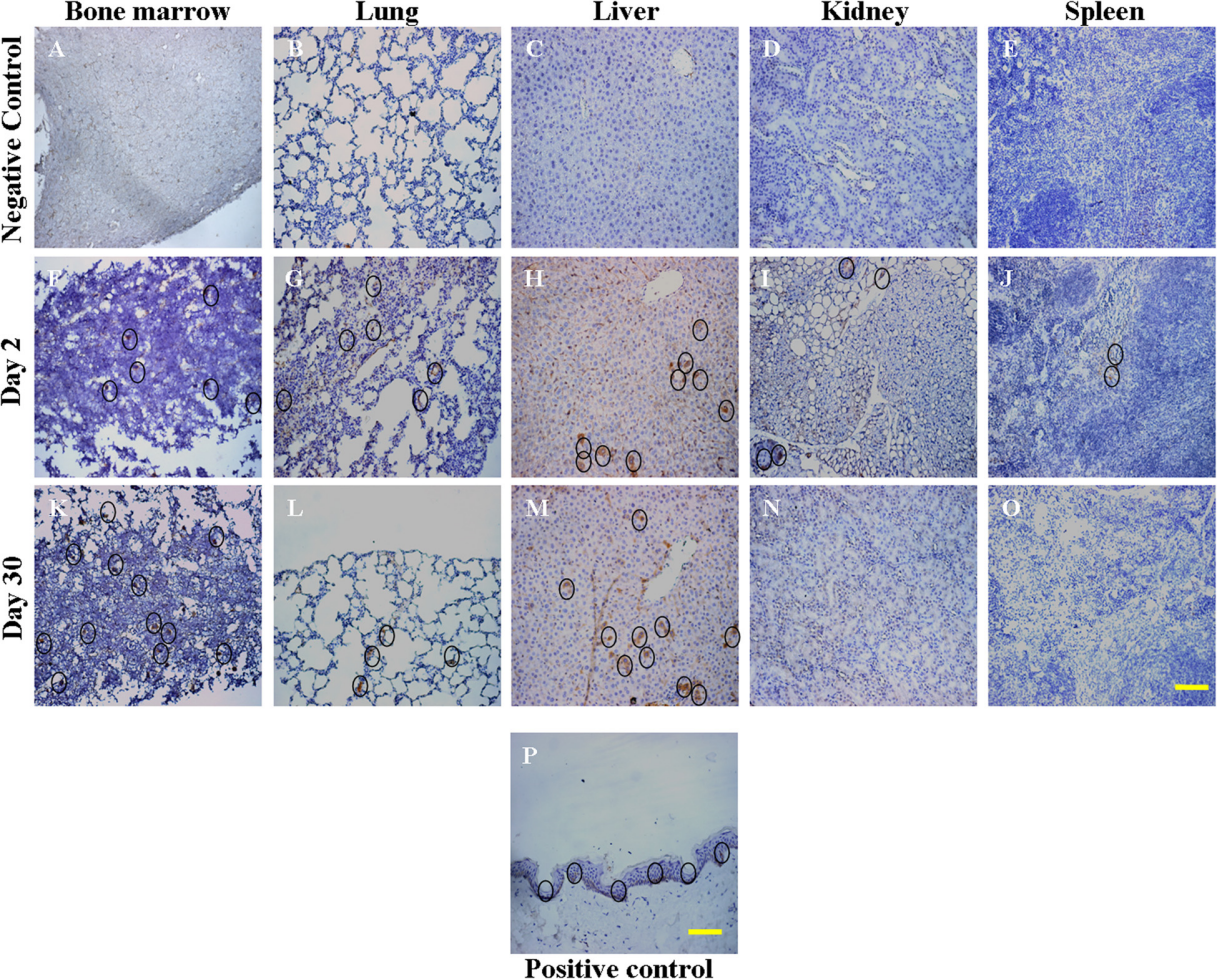
Immunohistochemical staining of infused human cells. Sections of human mitochondria were analyzed in bone marrow, lung, liver, kidney and spleen at day 1 (negative control group), day 2 and day 30 after human PDB-MSCs infused. No human cells were found in negative control group (**a**-**e**). At day 2 after ^14^C-TdR labeled PDB-MSCs infusion, PDB-MSCs were found in bone marrow, lung, liver, kidney and spleen (**f**-**j**). After 30 days,PDB-MSCs were found in bone marrow, lung, liver but not kidney and spleen (**k**-**o**). Human skin sections were stained as positive control (**p**). Scale bar = 100μm

Anti-human mitochondria antibody was used to detect the PDB-MSCs in the organs of nude mice. As shown in Fig. 4, negative result was found in control group (Fig. 4a-e); at day 2 after injection, human cells were found in bone marrow, lung, liver, kidney and spleen, which was coincident with the radioactivity analysis results (Fig. 4f-j). Positive staining was only found in bone marrow, lung and liver on day 30 (Fig. 4k-o). Thus, the same bio-distribution trend of PDB-MSCs in nude mice *via* IV injection was obtained after radioactivity and immunohistochemistry analysis.

## Discussion

In this study, it was determined that PDB-MSCs labeled with 1μCi ^14^C-TdR did not have alterations in biological characteristics, and that after IV injection in nude mice, human PDB-MSCs were found to be presented in bone marrow, lung, liver, spleen and stomach.

At present, MRI, scintigraphic tracking, quantum dots (QDs), reporter gene labeling and optimal imaging, have been widely used for cell tracking. MRI is the most widely used due to its safety and the capacity of 3-dimensional observation [17]. But several drawbacks were existed [18, 19]. For example, MRI cannot distinguish iron-labeled cells from the iron particles; second, the low sensitivity of MRI in clinical trails should be improved by up-regulating the magnetic field strengths, however, the toxic effects of MRI contrast agents on stem cells cannot be ignored [7, 19–22]. Another method for cell tracking is scintigraphy using radio-labeling materials: ^111^In-oxine, ^64^Cu-PTSM and ^124^I-FIAU, which can be visualized by positron emission tomography (PET) or single photon emission computed tomography (SPECT) and mainly applied in whole-body bio-distribution analysis [8, 19, 23–29]. This method is relatively highly sensitive and quantitative, however toxic effects on cell viability and photon attenuation by tissue should be considered [25, 30]. Also, QDs and reporter gene labeling induce cytotoxicity and require genetic modification or perturbation; additionally, quantification is difficult to be achieved [18]. Real time PCR (RT-PCR) is a valid alternative for the detection of human cells after xenografting, and had been utilized for detecting MSCs [28, 31–35] in mouse, rabbit and sheep models [33, 36]; but this technique is a little bit complex and is not efficient enough to pick up small cell numbers from the large organs.

In this work, ^14^C-TdR in the culture medium can be absorbed and incorporated in newly-synthesized DNA during cell replication, thus making it a stable tracer. As the aim of our research was to find a long-term, stable and quantitative method for PDB-MSCs tracking, hence comparison of the sensitivity of ^14^C-labeling method with other tracking methods was not performed, which should be investigated in future study.

After IV injection for 24 h, radioactivity was mainly presented in lung, kidney and spleen, which was consistent with the findings from other groups after IV injection of MSCs [26–28]. It was reported that 99 % of intravenously injected MSCs were cleared from the circulation system within 5 mins [37], thus it was reasonable that the radioactivity in blood was not noted after 24 h in this study. No PDB-MSCs was found in the brain, heart and muscle; this result was conflicted with some previous findings [9, 26, 27, 34, 36] which might be related to the variety of animal models, sources of MSCs and the trackers. It was demonstrated that the liver possessed a large proportion of infused cells [8, 26–29], which was not observed in our study until 5 days after infusion. The average diameter of human PDB-MSCs is 20-25μm (data not shown) and the diameter of capillaries in lungs is approximate 10 ~ 20μm; therefore, most of the infused cells may accumulate in the filtering organs such as the lungs, liver and spleen. Since the lung is considered as the first organ after IV injection, many literatures suggested that majority of infused cells were initially trapped into lung for the first 24 h [9, 29] or earlier [27–29] after IV injection. Apart from the lung, bone marrow and spleen were detected with the highest radioactivity on day 30. The possible explanation was that bone marrow is the major stem cell niche which might be the preferential migration target for MSCs. Spleen acts as a blood filter and it is an important part of immune system which might continuously filter cell debris or other incomplete catabolism products with ^14^C, thus increasing its radioactivity. Further studies need to be done to clarify the migration mechanism of intravenously injected MSCs, which is the major limitation in our study that we should acknowledge.

## Conclusion

In summary, PDB-MSCs could be labeled with 1μCi ^14^C-TdR successfully without significant changes in the proliferation, differentiation ability, CFU, karyotype and cell cycle profile; after intravenous injection, most of the ^14^C-TdR-labeled PDB-MSCs were initially located in the lung, spleen, stomach, femur and bone marrow and then the cells were quickly cleared in the first 5 days after injection; a small number of PDB-MSCs were detectable in liver, lung and left femur at 180 days after injection. Even though the cell number kept decreasing, this labeling method allowed the determination of the cell number presented at different time points. Therefore, [2-^14^C], ^14^C-TdR may be a stable, long-term and quantitative cell tracker after MSCs implantation.

## Methods

### Cell isolation and culture

Human placenta samples were obtained from three healthy donor mothers with written informed consent. The study has been approved by the ethics committee of West China Hospital, Sichuan University. PDB-MSCs were isolated according to our previous report [16]. Briefly, decidua basalis was collected and washed in phosphate-buffered saline to remove residual blood. The samples were mechanically minced into small particles, and digested with 0.25 % trypsin (Gibco, USA) and 0.1 % collagenase IV (Invitrogen, USA) and 80U/ml DNAse I (Sigma, USA) for 30 mins at 37°C. Nucleated cells were concentrated by density gradient centrifugation (500 g for 30 mins), suspended in 5 ml complete medium containing Dulbecco’s modified Eagle’s medium-High Glucose (DMEM-HG, Gibco, USA) with 10 % v/v fetal bovine serum (FBS, Hyclone, South America) and 1 % penicillin/streptomycin, and incubated in a 25 cm^2^ culture flask at 37 °C with 5 % CO_2_. After 70–80 % confluence, cells were passaged at a dilution of 1:3. Cells from the 4th passage were used in the subsequent assays.

### ^14^C-TdR labeling of PDB-MSCs

To determine the optimal labeling condition, 3 × 10^5^ PDB-MSCs were treated with different concentrations of ^14^C-TdR (PerkinElmer, Boston) (0.2μCi, 1μCi, 5μCi) and incubated for 72 h at 37°C with 5 % CO_2_. After incubation, cells were washed with phosphate buffer saline (PBS, pH = 7.4) twice and harvested by 0.25 % trypsin/ethylene diamine tetraacetic acid (EDTA); the radioactivity of ^14^C in labeled PDB-MSCs was measured by liquid scintillator counter (Beckman, USA) to estimate the uptake of each concentration.

### Cell proliferation

Cell proliferation was assessed using CCK-8 Kit (Dojindo, Japan). Briefly, 2 × 10^3^ PDB-MSCs and ^14^C-TdR labeled PDB-MSCs with four concentrations, were plated in 96-well. Cell viability was monitored on days 0, 1, 3, 5 and 7, respectively. One hour after the addition of 10μl CCK-8 in 100μl culture medium optical density was determined using a spectrophotometer at 490 nm with background correction at 630 nm.

### Cell differentiation

To analyze the effect of ^14^C-TdR labeling on the multi-potency of PDB-MSCs, osteogenic and adipogenic differentiation were performed in the unlabeled group and the 1μCi ^14^C-TdR labeled group. To induce osteogenic differentiation, radiolabeled and unlabeled PDB-MSCs were seeded in 6-well plates at a density of 5 × 10^3^ cells/well and cultured with osteogenic medium (complete medium with 10mM β-glycerol phosphate (Sigma, Switzerland), 0.1μM dexamethasone (Sigma, USA) and 50μg/ml ascorbate-2 (Sigma, USA)) for 21 days. The medium was changed every 3 days. After induction, cells were fixed with 75 % ethanol for 20 mins, washed with PBS twice and stained with Alizarin red solution (Sigma, USA) for 30 mins.

For adipogenic induction, labeled and unlabeled cells were plated at a density of 5 × 10^3^ cells/well and induced in the adipogenic medium consisting of 0.5μM isobutyl-methylxanthine (Sigma, USA), 0.25μM dexamethasone (Sigma, USA), 10μM insulin (Sigma, USA) and 50μM indomethacin (Sigma, Germany). The medium was replaced every 3 days. After 8 days, cells were fixed with 10 % formalin and stained for 30 mins with Oil red O solution (Sigma, USA) to visualize lipid vacuoles.

### CFU assay

For the analysis of colony forming ability, labeled and unlabeled PDB-MSCs were plated at a density of 80 cells/cm^2^ and cultured in completed medium. After 8 days, cells were washed with PBS, fixed with methanol for 5 mins and stained with 0.1 % crystal violet (Sigma, USA) solution for 30 mins at 37 °C. The number of MSCs clones was counted using the Image-Pro Plus 6.0 software (Media Cybernetics, USA).

### Karyotype analysis

For karyotype analysis, ^14^C-labeled PDB-MSCs were treated with 10μl/mL colcemid (Gibco, USA) for 2.5 h, added to 1ml 0.075M KCl solution for 20 mins and then fixed in 3ml Carnoy fixative for 30 mins at 37 °C. The cell specimens were made by standard air drying method. The G-band of labeled PDB-MSCs was analyzed by Metafer 4 Scanning System and the Ikaros Karyotype System (MetaSystems, USA).

### Cell cycle analysis

For cell cycle analysis, 2 × 10^6^ labeled and unlabeled PDB-MSCs were suspended in 70 % alcohol and stored at −4 °C overnight. After centrifugation and washing with PBS, cell pellets were incubated with 500μl staining solution containing 50 μg/ml PI (Sigma, USA), 0.1 mg/ml RNase A (Sigma, UK) and 0.05 % Triton X-100 for 30 mins in the dark at −4 °C. DNA index was measured by NovoCyte Flow Cytometer (ACEA Biosciences, China) and data were analyzed by NovoExpress analysis software (ACEA Biosciences, China).

### *In vivo* infusion and bio-distribution

All animal experiments were approved by the Institutional Animal Care and Use Committee of Sichuan University, and were carried out following Committee regulations and the Principles of Laboratory Animal Care formulated by the National Society for Medical Research. Four week-old female BALB/C nude mice (n = 21, purchased from Chengdu Dashuo Laboratory Animal Limited Company), used in this study, were randomly divided into 7 groups. In the experimental groups, each mouse was injected with approximately 1 × 10^6^ 1 μCi ^14^C-TdR labeled PDB-MSCs *via* caudal vein and unlabeled PDB-MSCs served as the control group.

Mice injected with labeled PDB-MSCs were sacrificed on days 1, 2, 3, 5, 30 and 180; mice injected with unlabeled PDB-MSCs were sacrificed on day 1. Organs including heart, liver, kidney, stomach, spleen, lung, blood, brain, left femur muscle, and the bone marrow from left femur (along with the femur itself) were harvested. Before the radioactivity assay, each organ was weighed and chemically digested with 100μl 30 % H_2_O_2_ (Kelong, Chengdu) and 200μl 60 % perchloric acid (Kelong, Chengdu) for 30 mins at 80 ~ 90 °C. During sample processing, the left femur containing both bone and the bone marrow was used *en bloc* since it was difficult to accurately weigh them. LS 6500 Scintillation Counter (Beckman, USA) was used to assess the radioactivity of each organ. Part of liver, lung and spleen, right femur and right kidney were prepared for immunohistochemical staining on day 2 and day 30, nude mice injected with PBS (pH = 7.4) were served as negative control group.

### Immunohistochemistry

For immunohistochemical staining, samples were fixed with 4 % paraformaldehyde for 48 h at room temperature. Bone samples were decalcified for 3 weeks in 10 % EDTA (pH 7.4) with weekly changes, and then all the samples were embedded in paraffin. Anti-human mitochondria antibody (1:400; ab92824, Abcam, Cambridge, UK) were used for detecting human cells in nude mice. After dewaxed and rehydrated, sections(4μm) were blocked with 3 % hydrogen peroxide for 15–20 mins at room temperature, followed by incubation with pepsin solution digest-all (Invitrogen, USA) for 20 mins at room temperature for bone sections antigen retrieval. Other organs sections were placed in trisodium citrate buffer (pH = 6.0) for 40 mins at 95 °C. After naturally cooled to room temperature, the sections were washed with PBS for 3 times and incubated with the primary antibody solutions overnight at 4 °C. Sections were rinsed with deionized water and then PBS, followed by 30 mins incubation with horse reddish peroxidase working solution (Gene Tech, Shanghai) at room temperature for 30 mins. After washed, 50-100μl 3,3′-diaminobenzidine (DAB) (Gene Tech, Shanghai) working solution was used for 5–10 mins at room temperature. The sections were then lightly counterstained with hematoxylin.

### Statistical analysis

All data were expressed as mean ± SD. Statistical analysis was performed using SPSS17.0 software (SPSS, USA). Results were analyzed with Student’s *T*-test and *P* < 0.05 were considered as statistically significant.

## Abbreviations

MSCs: Mesenchymal stem cells
PDB-MSCs: Human placental deciduas basalis derived MSCs
C-TdR: Carbon radioisotope labeling thymidine
MRI: Magnetic resonance imaging scans
QDs: Quantum dots
^3^H-TdR: Tritiated thymidine
dpm: Disintegrations per minute
CFU: Colony forming units
SD: Standard deviation
PET: Positron emission tomography
SPECT: Single photon emission computed tomography
RT-PCR: Real time PCR
DMEM-HG: Dulbecco’s modified Eagle’s medium-High Glucose
FBS: Fetal bovine serum
PBS: Phosphate buffer saline
EDTA: Ethylene diamine tetraacetic acid
DAB: 3,3′-diaminobenzidine
